# Supplementary material for: Enhanced Soil Fertility and Carbon Dynamics in Organic Farming Systems: The Role of Arbuscular Mycorrhizal Fungal Abundance
Source: J Fungi (Basel). 2024 Aug 24;10(9):598. doi: 10.3390/jof10090598 (PMC11433305; doi:10.3390/jof10090598)
Supplement: Supplementary file 1 [file jof-10-00598-s001.zip › jof-3101538-supplementary.pdf]

**TableS1.** Description of sampling site

| Region | Crop | Agricultural practice | Latitude | Longitude |
|---|---|---|---|---|
| Chuncheon | Maize | Conventional | 37.899999 | 127.666678 |
| | Maize | Conventional | 37.898923 | 127.665843 |
| | Potato | Conventional | 37.914142 | 127.726623 |
| | Potato | Conventional | 37.914843 | 127.751245 |
| | Potato | Organic | 38.021974 | 127.641835 |
| Goesan | Maize | Conventional | 36.833151 | 127.848342 |
| | Maize | Organic | 36.838515 | 127.863921 |
| | Maize | Organic | 36.839694 | 127.867614 |
| | Pepper | Conventional | 36.860423 | 127.835643 |
| | Pepper | Conventional | 36.866741 | 127.829933 |
| | Pepper | Organic | 36.855834 | 127.840515 |
| | Pepper | Organic | 36.855425 | 127.839889 |
| Gunsan | Maize | Conventional | 35.994813 | 126.789254 |
| | Maize | Organic | 35.993227 | 126.801392 |
| | Pepper | Conventional | 35.928168 | 126.688953 |
| | Pepper | Conventional | 35.914836 | 126.757234 |
| | Pepper | Organic | 35.941116 | 126.856357 |
| Hoengseong | Maize | Conventional | 37.596668 | 128.224892 |
| | Maize | Conventional | 37.569753 | 128.156557 |
| | Maize | Organic | 37.595546 | 128.224473 |
| | Maize | Organic | 37.595433 | 128.224426 |
| | Potato | Conventional | 37.592332 | 128.165932 |
| | Potato | Conventional | 37.592974 | 128.166945 |
| | Potato | Conventional | 37.592949 | 128.165963 |
| | Potato | Conventional | 37.592562 | 128.166548 |
| Wonju | Maize | Conventional | 37.176233 | 127.835422 |
| | Maize | Conventional | 37.178956 | 127.834714 |
| | Maize | Organic | 37.154338 | 127.829431 |
| | Maize | Organic | 37.250837 | 127.915531 |
| | Pepper | Conventional | 37.252619 | 127.914674 |
| | Pepper | Conventional | 37.322458 | 127.890343 |
| | Pepper | Organic | 37.157647 | 127.84955 |
| | Potato | Conventional | 37.174813 | 127.845732 |
| | Potato | Conventional | 37.158733 | 127.857867 |
| | Potato | Organic | 37.186212 | 127.834267 |
| | | Organic | 37.433303 | 127.987948 |

| | | | | |
|---|---|---|---|---|
| Yeongju | Maize | Conventional | 36.851525 | 128.708854 |
| | Maize | Conventional | 36.849634 | 128.710462 |
| | Pepper | Conventional | 36.847544 | 128.711117 |
| | Pepper | Conventional | 36.851524 | 128.708845 |
